# Effect of zinc on oropharyngeal mucositis in children with acute leukemia undergoing chemotherapy

**DOI:** 10.4317/medoral.23798

**Published:** 2020-10-09

**Authors:** Rosaura Gutiérrez-Vargas, Miguel Ángel Villasis-Keever, Javier Portilla-Robertson, Ivan De Jesús Ascencio-Montiel, Marta Zapata-Tarrés

**Affiliations:** 1 Program Master and Doctor of Medicine, Dentistry and Health Sciences. National Autonomous University of Mexico; 2 Research Unit in Analysis and Synthesis of Evidence. High Specialty Medical, Hospital of Pediatrics. XXI Century National Medical Center. Mexican Social Security Institute; 3 Coordination of Oral Pathology, Division of Graduate Studies, Faculty of Dentistry, National Autonomous University of Mexico; 4 Coordination of Epidemiological Surveillance, Mexican Social Security Institute; 5 Department of Medical Oncology, National Institute of Pediatrics, Health Secretary. Research Coordination, Fundación IMSS, A.C.

## Abstract

**Background:**

Oropharyngeal mucositis (OM) is one of the main side-effects of oncological therapy. There is no treatment to prevent its occurrence, but some zinc-based therapies have been proven to help in decreasing its intensity. The objective of this study was to determine the effect of zinc in OM in children with acute leukemia in the early stages of oncological treatment.

**Material and Methods:**

This quasi-experimental study evaluated OM in 2 groups (control group: conventional hospital management, and experimental group: administration of 50 mg of zinc gluconate daily plus conventional hospital management). OM severity was recorded at a two-month follow-up.

**Results:**

Forty-nine patients (26 in the control group and 23 in the experimental group) were included. The mean age of the patients was 11.1 ± 2.7 years; 65.3% had a diagnosis of pre-B acute lymphoblastic leukemia. The incidences of OM in the control group and the experimental group were 46.2% and 26.1%, respectively, but the difference was not significant. Based on a negative binomial regression model, females had, on average, 1.5 more days with OM (*p* = 0.002), and patients assigned to the experimental group had, on average, 2 less days with OM than the control group (*p* = 0.001). The pain score was higher in the control group (*p* = 0.0009), as was the mean score on the WHO scale (*p* = 0.0012).

**Conclusions:**

Zinc facilitated a reduction in the severity and duration of OM; further studies focusing on children are needed to confirm the effects of this trace element.

** Key words:**Oropharyngeal, mucositis, zinc, chemotherapy, children, leukemia.

## Introduction

The main oncological treatments in case of children include chemotherapy, radiotherapy, and hematopoietic stem cell transplantation (HSCT). Chemotherapy has a cytotoxic effect that eradicates malignant cells without discriminating against tissues with high cell division rates, such as the oral and gastrointestinal epithelium (1-5). It can also cause complications such as myelosuppression, hepatic or renal disorders and oropharyngeal mucositis (OM) (6). OM is defined as erythema and edema of the mucosa with progression to ulcers. Patients who also present with neutropenia and thrombocytopenia have an increased risk of oral bleeding and infections, which can lead to septicemia (2,7-9). OM involves pain, difficulty in swallowing and speaking, malnutrition, functional status deterioration, longer hospital stay, increased economic costs and decreased quality of life (3,4,7,8,10-13). In the pathophysiology of OM, 5 phases have been described in which a variety of cells are involved (12-14). DNA damage in epithelial basal cells causes keratinocyte growth factor loss. The production of reactive oxygen species (ROS) activates nuclear transcription factor kappa B, which upregulates tumor necrosis factor-α, IL-6 and IL-1 and leads to the apoptosis of fibroblasts and endothelial cells, thereby causing epithelial lesions. Also, bacteria activate macrophages, thus increasing the production of proinflammatory cytokines; subsequently, epithelial proliferation occurs until healing (10,11,14-16).

OM usually occurs between the 5th and 10th days after chemotherapy, resolving between days 7 and 14, given bone marrow suppression and infections do not occur (5,11,15), which is likely because the immune system of these patients is not effective at Fighting infections (16). Previous studies have reported that 80% of patients undergoing radiotherapy suffer from OM; this Figure increases to 90–100% in case of simultaneous chemotherapy, while it occurs in 20-80% of patients undergoing chemotherapy solely. In the latter case, the rate can increase to more than 90% in children under 12 years of age, and in those using HSCT, the OM occurrence rate can be >75% (1,6,10,11,13,15,17,18). There are different OM grades. The most widely used evaluation scale for defining OM severity is that of the World Health Organization (WHO), which classifies OM into 5 grades (0-4) (2,15,19).

Several studies suggest that the occurrence of OM is mainly associated with cytotoxic type and patient characteristics, being a frequent and difficult-to-resolve complication, despite the development of different therapies (3). The Multinational Association of Supportive Care in Cancer/International Society of Oral Oncology (MASCC/ISOO) has indicated that oral care (brushing and flossing) has a beneficial effect in the form of decreased oral bacteria (10,15,19). Cheng *et al*. reported the effectiveness of oral care in children, demonstrating a reduction in the incidence of OM (20). Low-level laser therapy has also been recommended in this context; however, it requires specialized equipment and training, and in children, cooperation is necessary (10,12). Topical ice application (cryotherapy) in the oral mucosa induces vasoconstriction and decreases exposure to the cytostatic agents, mainly 5-fluorouracil and melphalan; however, evidence of the benefits in pediatric patients is weak due to variances in chemotherapy type and treatment adherence, while results for methotrexate and etoposide are not conclusive (10-12,15).

Morphine or doxepin rinses have also been useful for pain (9,10). Anesthetic solutions with diphenhydramine, viscous lidocaine, bismuth subsalicylate, and corticosteroids are alternatives, but no significant improvement has been observed as a result of their use (11,15). Palifermin (keratinocyte growth factor-1) supports cell proliferation and helps in the prevention of OM; however, its efficacy and toxicity have not been demonstrated in children (9-12,18,19). Zinc is an antioxidant trace element essential for tissue repair, immune and anti-inflammatory functions and resistance to infections (9,17,18,21,22). It is a cofactor for DNA synthesis and important in cell proliferation (18,23) and may increase the gastrointestinal epithelial barrier function, thus decreasing cell death and detachment (3,23). For the above reasons, it can reduce the incidence and severity of OM during chemotherapy (3). The MASCC/ISOO suggests using zinc in oral cancer therapies (10,19). The recommended doses in children and adolescents range from 10 to 15 mg/day. However, for therapeutic purposes, the dose can be increased to 22.5 mg of elemental zinc/100 mg if administered as zinc sulfate, or to 30 mg or 80 mg elemental zinc/100 mg if administered as zinc acetate or zinc oxide, respectively (9). The objective of this study was to determine the effect of zinc in OM in children with acute leukemia in the early stages of oncological treatment.

## Material and Methods

This quasi-experimental study included all patients aged 8 to 16 years with a recent diagnosis of acute lymphoblastic leukemia (ALL) or acute myeloblastic leukemia (AML) who began their first oncological treatment at the National Pediatrics Institute (Instituto Nacional de Pediatría - INP) from 2016 to 2018. Patients who had previously undergone oncological treatment and showed psychomotor delays were excluded. Parents who agreed to participate signed an Informed Consent (IC) form, and children below 12 years of age signed an Informed Assent form. Children above 12 years of age were also asked to sign the IC form together with their parents.

All patients and parents voluntarily agreed to participate in the study. We instructed them to read and we explain all procedures, and we indicated that they could withdraw from a follow-up at any time during the study, without any impact on their medical treatment. The study was approved by the INP research and ethics committee. The sample size was the total population available. Because the recruitment was slow due to the inclusion criteria and having a research period of two years, the researchers decided to perform an intermediate analysis. The treatment allocation was done in blocks using sealed envelopes to have a balance in the number of participants in each group. The allocation of the group in sealed envelopes was carried out to identify and separate the effects of the treatments from the rest of the factors that affect the dependent variable.

The initial evaluation was performed before the patients started chemotherapy; Subsequently, evaluations were carried out every three days until they had completed 51 days of chemotherapy. With a total of 17 evaluations in each group, each evaluation included a data collection sheet that recorded crucial information such as the evaluation number, sex, age, diagnosis, level of neutropenia, chemotherapy protocol, use of methotrexate, oral hygiene (using the O'Leary index), oral pain (using a linear visual analogue scale (VAS), where 0 = no pain and 10 = maximum pain), day of onset of OM, days with OM and evaluation of the severity of OM with the WHO scale. Both groups were trained in the proper techniques for brushing their teeth and rinsing their mouth (Table 1); all participants were asked to report any adverse reactions.

Table 1 describes the intervention by group. The control group received the treatment that the hospital provided, and if OM was present, rinses with Philadelphia solution were indicated (in this study, the solution was prepared as a mixture of 30 ml of aluminum-hydroxide-based antacid (3.7 g), magnesium hydroxide (4.0 g), 30% simethicone emulsion equivalent to 0.5 g of dimethicone, vehicle, qs for 100 ml and 30 ml of diphenhydramine, a first-generation antihistamine). The experimental group received the treatment that the hospital provided and 50 mg of zinc gluconate daily, equivalent to 7 mg of elemental zinc, a dose similar to that recommended in children. Zinc gluconate (zinc bound to gluconic acid) was chosen to decrease the irritating effect on the gastric mucosa. Zinc gluconate is an over-the-counter food supplement that does not require a prescription, so it was not necessary to register the study with the federal commission for protection against health risks. Both groups were instructed to rinse with baking soda after each meal, ~40 seconds.

The most important outcome variable was OM severity (using the WHO scale) and days with OM (from the time when OM was detected to recovery of the oral epithelium). OM severity was evaluated in artificial light, with all physical protection barriers in place, by 2 examiners who had been previously trained and standardized (weighted Kappa coefficient = 0.72). In addition, the other variables analyzed were sex, age, diagnosis, neutropenia, chemotherapy protocol, type of chemotherapy, oral hygiene and oral pain.

All materials were given to each of the participants. To verify response and adherence to treatment, cards were made for each group, and the number of zinc Tablets and the consumption of materials for each group were monitored.

Statistical analysis. The qualitative data are presented as absolute numbers and percentages, while the quantitative data are presented as the mean and standard deviation (SD). Fisher’s exact test, chi-squared test, Student’s t-test, and analysis of variance (two-way ANOVA) were used to compare the groups with respect to OM grade and the VAS for oral pain. To evaluate the association between days with OM and the assigned group, a multivariate negative binomial regression model was used, adjusting for sex, age, neutropenia, diagnosis, chemotherapy, use of methotrexate and oral hygiene. The data were analyzed with STATA version 13.0.

**Table 1:**
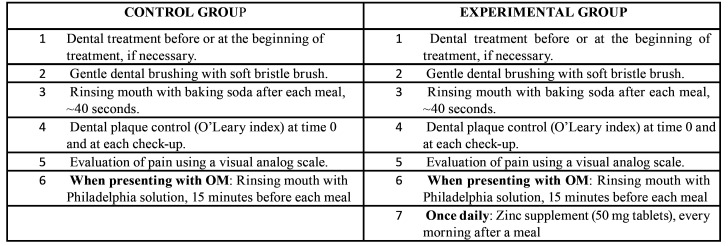
Description of the intervention in each study group.

## Results

**Figure 1:**
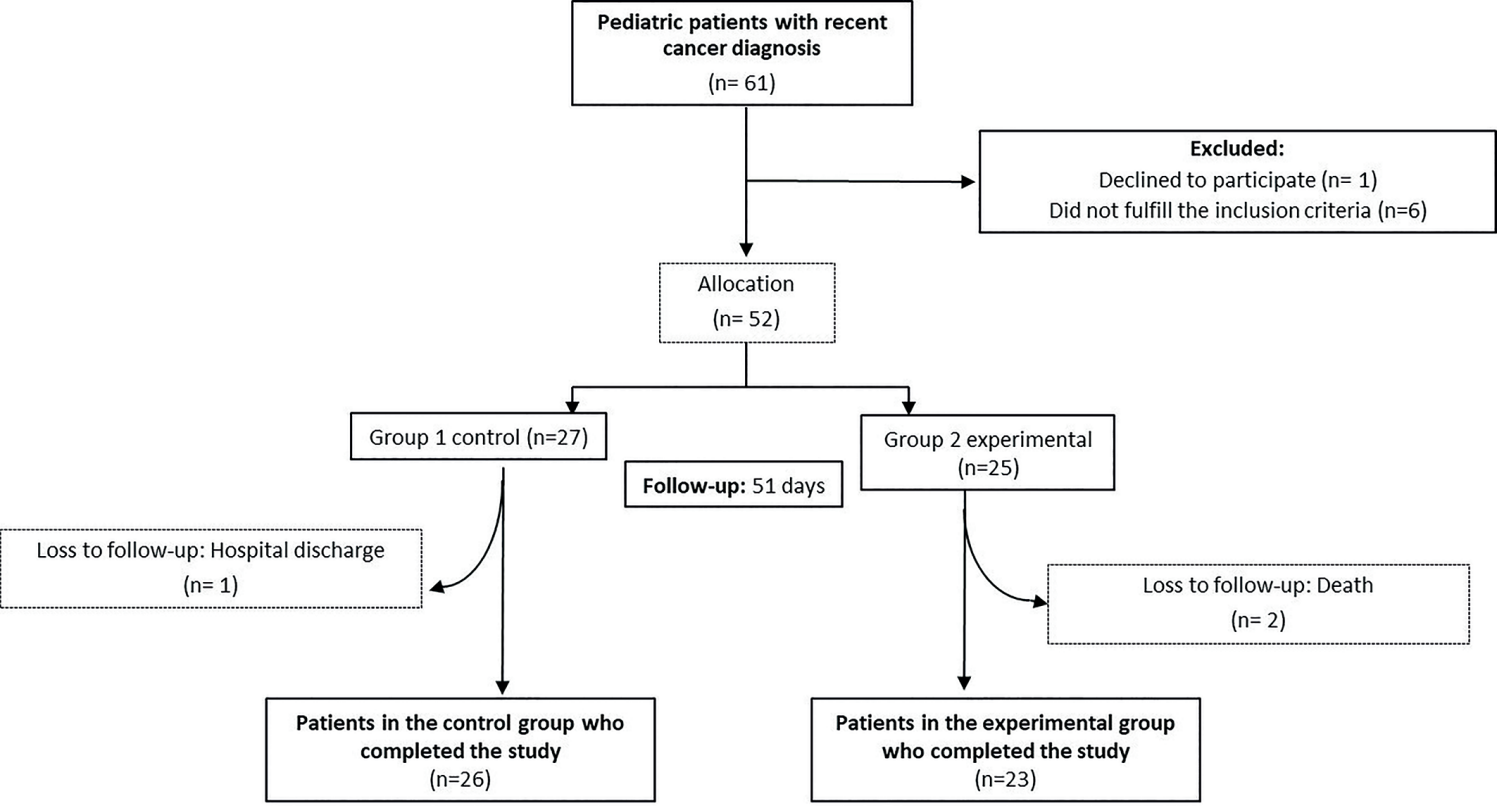
Flowchart of patients who participated in the study.

The study included 61 patients, out of which 52 were divided into two groups according to the inclusion criteria and there were three losses during the follow-up (Fig. 1). In the 49 patients, the mean age was 11.1 ± 2.7 years. Of these, 59.2% were male, and 65.3% were diagnosed with pre-B ALL. In the baseline comparison, the groups did not show significant differences in sex, age, diagnosis, neutropenia, chemotherapy protocol or oral hygiene (Table 2).

During follow-up, a greater number of days with OM (14.2 ± 21.1 vs. 5.1 ± 11.2, *p* = 0.072) and a higher incidence of OM (46.2% vs. 26.1%, *p*= 0.146) were observed in the control group compared with the experimental group; however, the differences were not significant (Table 3). We used a negative binomial regression model for days with OM (Table 4).

**Figure 2:**
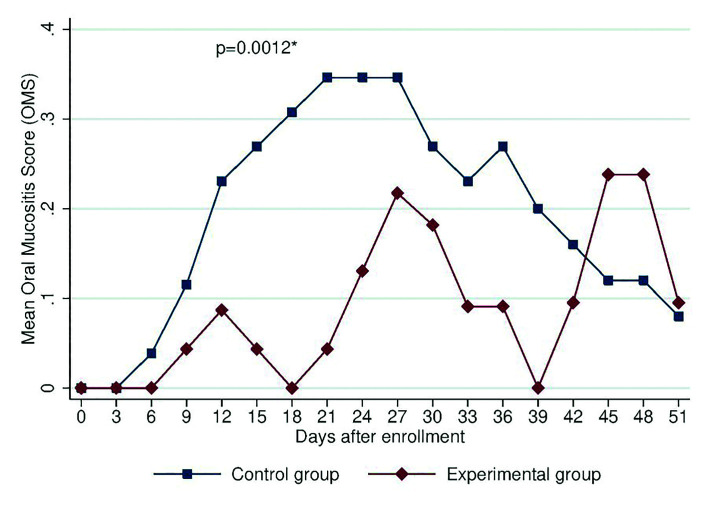
Mean score on the WHO OM scale by allocation group during the evaluation.

**Figure 3:**
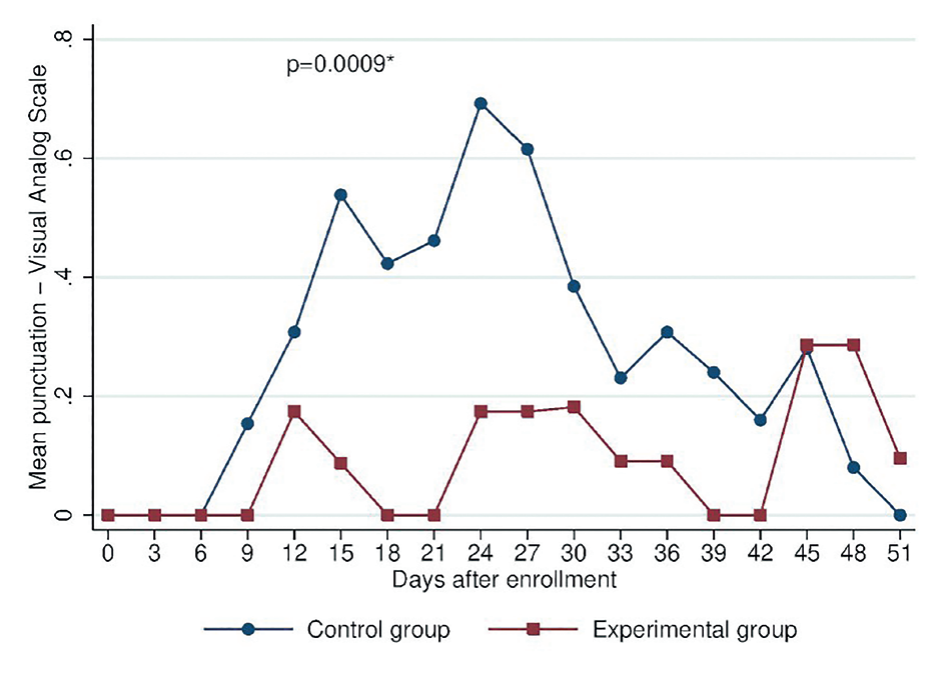
Mean pain score obtained using a visual analog scale, at the different days after enrollment, for each group.

The experimental group had an average of 2 less days with OM than the control group (*p* = 0.001), and girls had an average of 1.5 more days with OM than boys (*p* = 0.002). With regard to the type of chemotherapy protocol, patients with 7+3+2, 7+3 and ADE had, on average, 1.5 less days with OM than patients with other protocols (*p* = 0.046). The mean OM severity and mean oral pain scores for the groups were significantly different (F = 10.56, *p* = 0.0012 and F = 11.12, *p* = 0.0009, respectively). As shown in Fig. 2 and Fig. 3, patients who received zinc had lower OM severity and associated pain, particularly between days 18 and 39.

**Table 2:**
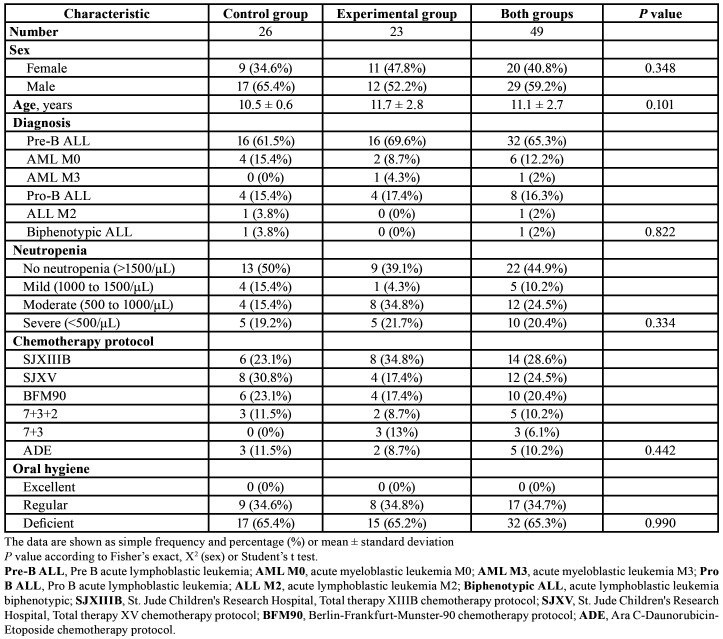
Demographic and clinical characteristics of the participants at the beginning of the study.

**Table 3:**
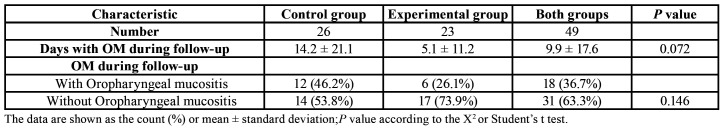
Clinical characteristics of study participants during follow-up.

**Table 4:**
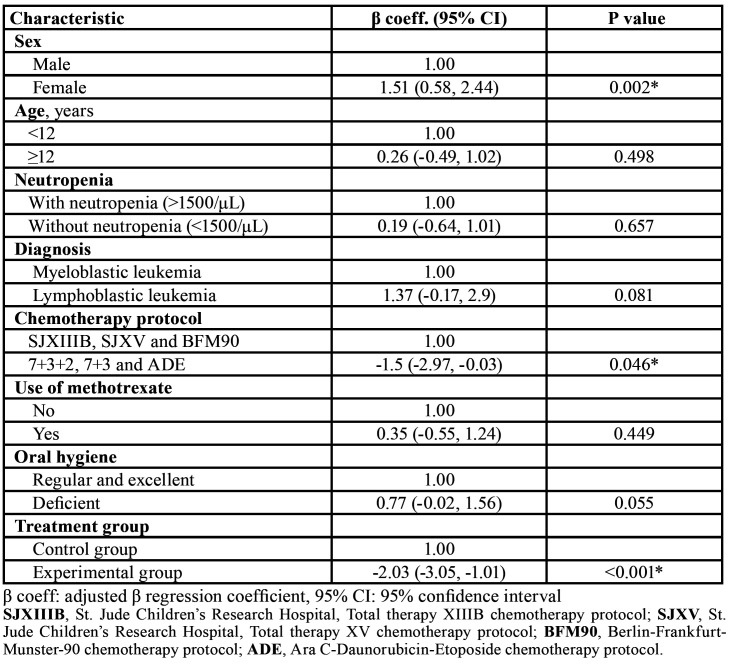
Multivariate analysis using negative binomial regression.

## Discussion

The results of this study demonstrate that zinc reduces OM severity (both in terms of pain and duration of disorder) in pediatric patients with leukemia undergoing chemotherapy; however, no significant difference was found in OM incidence between the experimental and control groups. Minimal requirements for children varied between 10 and 15 mg/day. We decided to implement for therapeutic purposes 50-mg zinc gluconate, equivalent to 7 mg of elemental zinc, a dose similar to that recommended in children. Furthermore, there are a few studies done on children that used zinc gluconate to decrease the effects of OM; most of the studies have been carried out with zinc sulfate in adults and considering that the desired effect was obtained with doses of 150mg/day, we considered a third of the dose for this study.

Shuai *et al*. described the anti-inflammatory and antioxidant effects of zinc, resulting from its ability to eliminate ROS, mediate the expression of COX-2 and inhibit the release of PGE2 (11), which may reduce mucotoxicity and explain why patients in this and other studies have benefited from zinc use (18).

Rambod *et al*. administered zinc sulfate (220 mg daily) to 44 leukemic adults undergoing chemotherapy and observed a difference in OM incidence between the experimental and control groups at 25.0% and 54.2% respectively (*p* = 0.01) (23). In comparison, in the present study, although there was also a lower incidence of OM in the group that took zinc (46.2% vs. 26.1%), the difference was not significant (*p* = 0.146). However, with respect to the number of days with OM, the time was shorter for the experimental group than for the control group (5.1 ± 11.21 vs. 14.2 ± 21.1, *p* = 0.072), which is consistent with the findings of Arbidi *et al*., who described that OM recovery was shorter in the zinc group than in a placebo group, but the difference was not significant (*p* = 0.13) (17).

Notably, other studies have not demonstrated the possible benefit of the use of zinc for countering OM. Mansouri *et al*. administered 440 mg of zinc sulfate daily (versus placebo to the control group) to blood cancer patients over 15 years of age undergoing HSCT and found no reduction in severity, differences in the day of onset and duration of OM in the experimental group (*p* >0.05) (18). Similarly, Tian *et al*. reported that zinc sulfate decreased neither the incidence (RR = 0.52; 95% CI: 0.17-11.64) nor the grade of OM due to chemotherapy (RR = 0.62, 95% CI: 0.11-3.56; RR = 0.70, 95% CI: 0.29-1.71), and that it was also not associated with pain reduction, late onset, decreased adverse events or a better quality of life compared to controls (3). Unlike these studies, a multivariate analysis of our data showed that the experimental group had, on average, 2 less days with OM (*p* = 0.001) from detection until epithelial recovery (beta coeff. -2.03; 95% CI: -3.05, -1.01, *p* <0.001) compared to the control group. The lack of consistency between the different studies was also illustrated by Chaytana *et al*. in a systematic review. They reported that 8 out of 10 studies favored zinc over placebo, and when performing the meta-analysis, they found an overall effect size of -0.89 (95% CI: -1.08, -0.70, *p* <0.00001) for the different zinc supplements used (9).

Another characteristic evaluated in our population was oral pain, with the experimental group having a lower mean score than the control group (F = 11.12, *p* = 0.0009), which is consistent with the findings of Rambod *et al*., who described a significant difference in the mean score for the subjective evaluation of OM (lesions, erythema, edema, pain and dysphagia) (F = 5.79, *p* = 0.01) and in the mean score on the WHO OM scale (F = 7.83, *p* = 0.007) (23).

Our data showed that the highest pain scores for the control group were found between days 15 and 30 (weeks 3 and 5) and at days 45 to 48, almost at the end of follow-up (weeks 6 and 7), in the experimental group. Similarly, Ribeira *et al*. reported, in a descriptive study of children with acute leukemia undergoing chemotherapy, that pain, mainly upon swallowing, had a greater frequency starting at week 8 (5). The OM scores were higher for both groups during the start of the consolidation phase—a finding partially consistent with the results reported by Curra *et al*. That is, OM did not occur during the induction of leukemia treatment but rather in the consolidation phase in 6.1% and in the maintenance phase in 8.2% (24) of the participants.

In this study, the chemotherapy protocols—7+3+2, 7+3 and ADE—used to treat AML were those that on average were associated with 1.5 less days with OM (beta coeff. -1.5; 95% CI: -2.97, -0.03, *p* = 0.046) compared with other protocols (SJXIIIB, SJXV and BFM90), which included methotrexate, cytarabine, 6-mercaptopurine, etoposide, daunorubicin and vincristine, some of which have been described as cytotoxic and highly associated with OM (25). Bishop *et al*. described that daunorubicin and etoposide led to the development of severe OM in 26% of patients who were more than 55 years old with acute nonlymphocytic leukemia (26).

In the present study, we tried to avoid interrupting the course of chemotherapy due to oral manifestations so that all patients with caries were treated before and during chemotherapy. Additionally, because no adverse clinical reactions to zinc were observed (rashes, vomiting, nausea not associated with chemotherapy), no side effects were monitored via blood analysis. Besides this observation, patients received chemotherapy during the intervention. Chemotherapy generates hemoglobin, leukocites, platelet changes, as well as, in a fewer degree, renal and hepatic transitory changes. This is the reason we decide not to report this information. The major limitations included the small sample size, blinding of participants was not carried out, and no placebo was used in the control group. Children with acute lymphoblastic leukemia during their treatment received steroids and more than eight chemotherapeutic agents. We considered that even if placebo usually does not provoke adverse events, the placebo is generally composed of sugar and secondary diabetes by steroid/L-asparaginasa is an important risk in these patients.

We also acknowledge the probable masking of pain due to the prescription of intravenous analgesics to decrease the pain due to routine medical procedures (venous canalization, bone marrow aspirates, placement of venous catheter, among others) that could decrease the symptomatology of the pain caused by OM, together with the application of the philaphelphia solution to control local pain. In this study, there was no need to indicate an opioid analgesic, nor was there any need to use a nasogastric tube, since OM grade 4 did not occur in either of the two groups; there were also no bacteremia due to OM. The analgesic doses were adjusted at the discretion of the treating physician. We reported three participants’ follow-up losses due to voluntary discharge and two due to death, owing to systematic complications due to cancer.

This study, designed to evaluate the effect of zinc on chemotherapy-induced OM in pediatric patients with acute leukemia, showed a reduction in OM severity and duration and associated pain; however, a larger sample size is needed to confirm the beneficial effects of this trace element. Initially, we worked with a sample size calculated with the formula to compare two proportions, according to the study by Cheng *et al*. (20), for a difference of 50% between the experimental group and the control group. We estimated 38 patients per group and made an intermediate analysis and significative differences were obtained, so we decided to stop recruitment.

Although it was not the main objective of the study, Kaplan-Meier overall survival estimates among groups was calculated. The overall survival was 100% (26/26) in the control group and 88.8% (23/25) in the experimental group. In the cause of the two deaths was not related to sepsis nor OM. Moreover, Kaplan-Meier survival estimates were not statistically significant among groups.

The treatment of a child or adolescent with cancer includes many drugs, complications and symptoms typical of the disease; therefore, it is difficult to conduct a study without confounders. There are not enough studies, in the context of children that describe the maximum dose of zinc and the optimal time of administration during oncological treatment to achieve clinical effectiveness in preventing or reducing OM severity. The great variety of studies that describe presentations, age groups and hematological conditions place zinc as an adjuvant in the prevention and treatment of OM with moderate evidence; however, because OM is a frequent complication that often interrupts oncological treatment, any therapy that presents significant clinical benefits without adverse effects should be implemented.
